# A Novel Nomogram to Predict Pathological Complete Response in Breast Cancer Patients and Identify Candidates Who Might Omit Surgery: A Large Cohort Study

**DOI:** 10.1002/cam4.71372

**Published:** 2025-11-08

**Authors:** Kaining Ye, Xuehong Liao, Weiping Yang, Jianming Weng, Yongliang Dai, Xiliang Chen, Yongjian Liu, Kaixin Du

**Affiliations:** ^1^ Department of Pathology Zhangzhou Affiliated Hospital of Fujian Medical University Zhangzhou China; ^2^ Department of Pathology Sapporo Medical University Sapporo Japan; ^3^ Department of Radiation Oncology Fujian Medical University Xiamen Humanity Hospital Xiamen China; ^4^ Department of Pathology Fujian Medical University Xiamen Humanity Hospital Xiamen China; ^5^ Department of Pathology Zhangzhou Traditional Chinese Medical Hospital Zhangzhou China

**Keywords:** breast cancer, complete response, nomogram, non‐surgery, SEER database

## Abstract

**Purpose:**

To establish and validate a nomogram for predicting pathological complete response (pCR) after neoadjuvant therapy (NAT) in breast cancer (BC) patients, aiming to identify subgroups potentially suitable for non‐surgical management.

**Methods:**

Between 2010 and 2015, 4402 BC patients (3037 surgery, 1365 non‐surgery) were extracted from the Surveillance, Epidemiology, and End Results (SEER) database, with external validation in 339 BC patients from our hospital. Logistic regression identified pCR predictors and a nomogram model was constructed. Propensity score matching (PSM) was applied to minimize the effect of the imbalance of the prognostic factors between the surgery group and the non‐surgery group.

**Results:**

Univariable and multivariable analyses revealed that age, marital status, T stage, N stage, differentiation grade, hormone receptor (HR) status, human epidermal growth factor receptor 2 (HER2) status and chemotherapy were significant predictors of pCR in the surgery group (all *p <* 0.05). The area under the receiver operating characteristic curve (AUC) for predicting pCR was 0.756 (95% CI: 0.736–0.776), 0.717 (95% CI: 0.681–0.754), and 0.744 (95% CI: 0.687–0.800) in the training, internal validation, and external validation sets, respectively. Non‐surgery patients were stratified into high (> 375), medium (162.5–375), and low (< 162.5) nomogram score groups, with 5‐year OS rates of 70.7%, 49.2%, and 29.2% (*p* < 0.05). After PSM, 358 pairs of patients were included to compare 5‐year OS between the surgery group and the non‐surgery group, and the results showed that the 5‐year OS of patients stratified by high‐score group, medium‐score group and low‐score group between the surgery group and the non‐surgery group were 85.4% vs. 72.8%, 78.3% vs. 59.8% and 79.3% vs. 20.0% (all *p* < 0.05).

**Conclusion:**

We successfully established a nomogram for pCR in BC patients. Based on this predictive model, patients with higher scores may represent potential candidates for non‐surgical therapeutic approaches, warranting further investigation in future studies.

## 1 Introduction

Breast cancer (BC) has become the most common malignant tumor among women [1]. While surgery remains the cornerstone of early‐stage BC treatment, its physical and psychological impacts are considerable [2]. With the continuous advancements in technology, including accurate genetic testing, advanced radiotherapy equipment, sensitive diagnostic tools, and more effective anti‐tumor drugs, some breast cancer patients may have the potential to avoid surgery. To our knowledge, there are several types of tumors that can be effectively treated with radical chemoradiotherapy, and in some cases yield better outcomes than surgery, such as nasopharyngeal cancer, cervical cancer and prostate cancer [3, 4, 5]. A “watch and wait” strategy has also been proposed for rectal cancer. This approach allows patients who achieve complete response after neoadjuvant therapy (NAT) to join the watch‐and‐wait group, thereby enabling some rectal cancer patients to avoid surgery and achieve a higher quality of life [6]. Several studies have demonstrated that BC patients who achieve pathological complete response (pCR) after NAT exhibit excellent survival rates [7, 8, 9]. For those pCR patients, omitting or delaying surgery and adopting a “watch and wait” approach may be an optional strategy. M. Morrow et al. also proposed that if pCR can be determined without surgery, these patients with a high local control rate may have the opportunity to avoid surgery by combining it with radiotherapy [10]. However, there are currently no prospective trials to confirm this conclusion. Therefore, developing a preoperative pCR prediction model is crucial for optimizing treatment decision‐making and informing future prospective trials.

The Surveillance, Epidemiology, and End Results (SEER) database, the largest tumor database globally, provides comprehensive information on tumors, outcomes, and responses to NAT since 2010 [11]. The nomogram is considered to be a reliable tool for predicting the prognosis of cancer patients [12]. We constructed a large‐sample nomogram using the SEER database to predict pCR after NAT and identified subgroups of BC patients who might achieve longer survival even without surgery.

## 2 Materials and Methods

### 2.1 The Surveillance, Epidemiology, and End Results (SEER) Database Analyses

**FIGURE 1:**
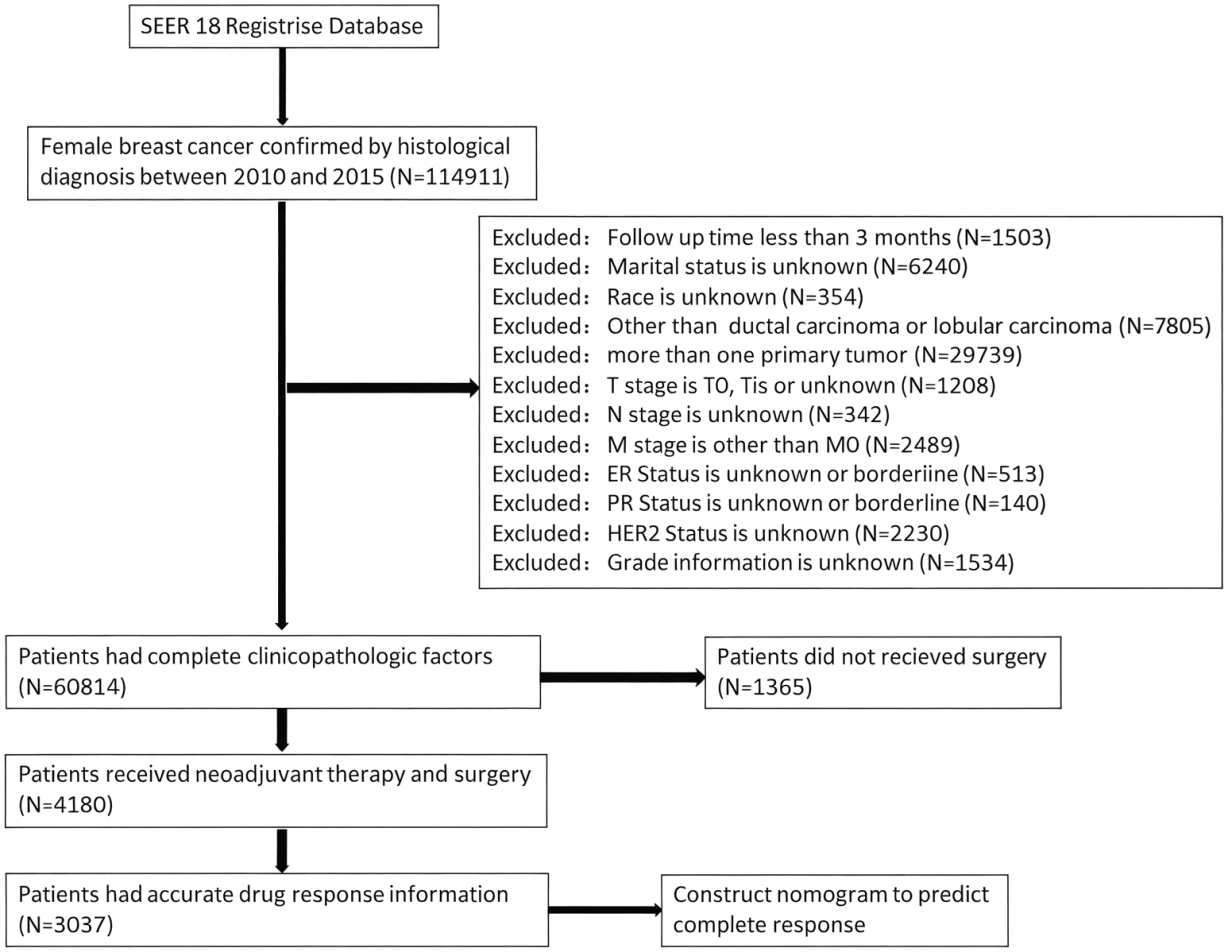
Flow diagram of patient selection from the SEER database.

We used SEER*Stat version 8.4.0 to retrieve the SEER database (serial number: 12068‐Nov2022). There were 114,911 female BC patients with histologically confirmed diagnoses from 2010 to 2015. We present the selection flowchart in Figure 1. The exclusion criteria were as follows: (1) Follow‐up less than 3 months; (2) Marital status was unknown; (3) Race was unknown; (4) Pathology was other than infiltrating ductal carcinoma (IDC‐O‐3 morphology codes 8500, 8521, and 8523), infiltrating lobular carcinoma (ILC codes 8520 and 8524), or components of both (code 8522.); (5) More than one primary tumor; (6) AJCC T stage was T0, Tis or unknown; (7) N stage was unknown; (8) M stage was other than M0.; (9) Estrogen receptor (ER) status is unknown or borderline; (10) Progesterone receptor (PR) status was unknown or borderline; (11) Human epidermal growth factor receptor 2 (HER‐2) status was unknown or borderline; (12) Grade information was unknown. As the data were obtained from a public database, informed consent was not required. In addition, treatment‐specific (surgery, chemotherapy and radiotherapy) characteristics were obtained. Among them, 3037 BC patients who had pCR information to NAT after surgery (surgery group) and 1365 patients who did not receive surgery were included in our study (non‐surgery group). No residual invasive carcinoma in the breast after NAT, or only residual in situ carcinoma, is classified as a pCR.

### 2.2 Data Preprocessing and Cohort Division

The dataset was randomly divided into a training set (70%) and an internal validation set (30%). The training set was used for model construction, and the internal validation set was used for performance evaluation. Data preprocessing included missing value imputation, normalization of continuous variables, and categorical variable encoding.

An independent external validation cohort from our hospital was used to further assess the generalizability of the model. This study retrospectively analyzed 339 BC patients who received NAT and underwent surgery between January 2017 and January 2024. The BC patients were included in the study with complete information on age, marital status, T stage, N stage, differentiation, HR status, HER‐2 status, histological subtype, and response to NAT treatment based on pathology. These patients were used to perform external validation of the model predicting pCR. Ethical approval from our hospital was obtained (2025‐LWB‐221).

### 2.3 Statistical Analysis

Statistical analyses were performed using SPSS 24.0 (IBM Corp., Armonk, NY). Differences between groups were assessed with a *p*‐value < 0.05 indicating significance. Kaplan–Meier analysis with log‐rank tests estimated OS. Logistic regression (univariate and multivariate) identified pCR predictors in the surgery group. A nomogram was developed using independent predictors, evaluated via receiver operating characteristic (ROC) curves and calibration plots (1000 bootstrap resamples). X‐tile software determined nomogram score cutoffs. The propensity score matching (PSM) ratio was set at 1:1 to minimize the differences between the surgery group and the non‐surgery group.

## 3 Results

### 3.1 Patient Characteristics

The characteristics, clinical features, and treatment strategies are summarized in Table 1. Of 4402 eligible BC patients (median follow‐up: 62 months, range: 3–119), 3037 underwent surgery and 1365 did not (Table 1). In the surgery group, 76.6% were ≤ 60 years, 60.6% were married, and 90.3% had invasive ductal carcinoma. T stages were T1 (16.9%), T2 (52.5%), T3 (20.1%), and T4 (10.5%); N stages were N0 (34.7%), N1 (45.9%), N2 (11.4%), and N3 (8.0%). Most (60.4%) had grade III/IV tumors. Hormone receptor status included ER+/PR+ (42.7%), ER−/PR− (38.2%), and HER2 was positive in 35.5%. Chemotherapy and radiotherapy rates were 96.6% and 71.5%, respectively. The majority of patients (90.3%) had invasive ductal carcinoma, with a pCR rate of 40.3% (1224/3037). In the non‐surgery group, 480 (35.2%) patients were ≤ 60 years and 498 (36.5%) patients were married. 438 (32.1%), 570 (41.8%), 180 (13.2%) and 177 (13.0%) patients were classified as stage T1, T2, T3 and T4, respectively. And 820 (60.1%), 442 (32.4%), 66 (4.8%) and 37 (2.7%) patients were classified as stage N0, N1, N2 and N3, respectively. The majority of patients were grade III or grade IV (35.5%, 485/1365). The proportions of ER+/PR+, ER+/PR−, ER−/PR+ and ER−/PR− were 68.4% (934/1365), 12.7% (173/1365), 1.5% (21/1365) and 17.4% (237/1365), respectively. The HER‐2 status was negative in 82.5% and positive in 17.5% of cases. The pathology of most patients (85.9%) was ductal carcinoma. Only 26.6% (363/1365) of patients received chemotherapy and 2.1% (29/1365) of patients received radiotherapy.

**TABLE 1:** Demographics of patients before and after propensity score matching.

Characteristic	Before matching			After matching		
	Surgery group (*N* = 3037)	Non‐surgery group (*N* = 1365)	*p*	Surgery group (*N* = 358)	Non‐surgery group (*N* = 358)	*p*
Age			< 0.001			0.754
≤ 60	2326 (76.6)	480 (35.2)		230 (64.2)	234 (65.4)	
> 60	711 (23.4)	885 (64.8)		128 (35.8)	124 (34.6)	
Marital status			< 0.001			0.577
Married	1840 (60.6)	498 (36.5)		184 (51.4)	198 (55.3)	
Single	640 (21.1)	269 (19.7)		84 (23.5)	77 (21.5)	
Other^a^	557 (18.3)	598 (43.8)		90 (25.1)	83 (23.2)	
Race			0.053			0.107
White	2134 (70.3)	994 (72.8)		236 (51.4)	261 (55.3)	
Black	505 (16.6)	227 (16.6)		79 (23.5)	59 (21.5)	
Other^b^	398 (13.1)	144 (10.5)		43 (25.1)	38 (23.2)	
Primary site			< 0.001			0.650
Central	115 (3.8)	88 (6.4)		19 (5.3)	24 (6.7)	
Inner	508 (16.7)	229 (16.8)		50 (14.0)	49 (13.7)	
Outer	1346 (44.3)	512 (37.5)		136 (38.0)	150 (41.9)	
Overlap	672 (22.1)	329 (24.1)		93 (26.0)	80 (22.3)	
Unknown	396 (13.0)	207 (15.2)		60 (16.8)	55 (15.4)	
AJCC T stage			< 0.001			0.550
T1	512 (16.9)	438 (32.1)		56 (15.6)	61 (17.0)	
T2	1594 (52.5)	570 (41.8)		185 (51.7)	167 (46.6)	
T3	612 (20.1)	180 (13.2)		75 (20.9)	79 (22.1)	
T4	319 (10.5)	177 (13.0)		42 (11.7)	51 (14.2)	
AJCC N stage			< 0.001			0.077
N0	1054 (34.7)	820 (60.1)		56 (40.2)	114 (31.8)	
N1	1393 (45.9)	442 (32.4)		185 (42.5)	184 (51.4)	
N2	347 (11.4)	66 (4.8)		75 (10.3)	33 (9.2)	
N3	243 (8.0)	37 (2.7)		42 (7.0)	27 (7.5)	
Differentiation grade			< 0.001			0.381
I	172 (5.7)	260 (19.0)		34 (9.5)	45 (12.6)	
II	1031 (33.9)	620 (45.4)		132 (36.9)	122 (34.1)	
III/IV	1834 (60.4)	485 (35.5)		192 (53.6)	191 (53.4)	
HR			< 0.001			0.368
ER+/PR+	1296 (42.7)	934 (68.4)		168 (46.9)	181 (50.6)	
ER+/PR−	504 (16.6)	173 (12.7)		60 (16.8)	48 (13.4)	
ER−/PR+	77 (2.5)	21 (1.5)		8 (2.2)	13 (3.6)	
ER−/PR−	1160 (38.2)	237 (17.4)		122 (34.1)	116 (32.4)	
HER2			< 0.001			0.628
Negative	1960 (64.5)	1126 (82.5)		244 (68.2)	250 (69.8)	
Positive	1077 (35.5)	239 (17.5)		114 (31.8)	108 (30.2)	
Histological subtype			< 0.001			0.242
Ductal	2742 (90.3)	1172 (85.9)		312 (87.2)	306 (85.5)	
Lobular	169 (5.6)	139 (10.2)		31 (8.7)	27 (7.5)	
Mix	126 (4.1)	54 (4.0)		15 (4.2)	25 (7.0)	
Chemotherapy			< 0.001			0.476
No	102 (3.4)	1002 (73.4)		61 (17.0)	54 (15.1)	
Yes	2935 (96.6)	363 (26.6)		297 (83.0)	304 (84.9)	
Radiotherapy			< 0.001			0.341
No	865 (28.5)	1336 (97.9)		325 (90.8)	332 (92.7)	
Yes	2172 (71.5)	29 (2.1)		33 (9.2)	26 (7.3)	

Abbreviations: HER‐2, human epidermal growth factor receptor 2; HR, hormone receptor.

^a^ Others included Divorced/Separated/Widowed/Unmarried.

^b^ Others included American Indian/Alaskan Native, Asian.

### 3.2 Univariate and Multivariate Logistic Regression Analysis

Univariable logistic regression (Table 2) showed age (*p* < 0.001), marital status (*p* < 0.001), primary site (*p* = 0.001), T stage (*p* < 0.001), N stage (*p* < 0.001), grade (*p* < 0.001), HR status (*p* < 0.001), HER‐2 status (*p* < 0.001), histological subtype (*p* < 0.001), chemotherapy (*p* < 0.001) and radiotherapy (*p* < 0.001) were significantly associated with pCR. The multivariate logistic regression analysis identified younger age (*p* < 0.001), married (*p* = 0.004), earlier T stage (*p* < 0.001), lymph nodes without metastasis (*p* < 0.001), poor differentiation (*p* < 0.001), ER (−)/PR (−) (*p* < 0.001), HER‐2 (+) (*p* < 0.001) and receiving chemotherapy (*p* = 0.016) as independent pCR predictors.

**TABLE 2:** Univariate and multivariate logistic regression of variables predicting complete response to neoadjuvant therapy in surgery group.

Characteristic	Univariate analysis		Multivariate analysis	
	OR (95% CI)	*p*	OR (95% CI)	*p*
Age		< 0.001		< 0.001
≤ 60	Reference		Reference	
> 60	0.651 (0.546–0.777)		0.643 (0.523–0.790)	
Marital status		< 0.001		0.004
Married	Reference		Reference	
Single	0.797 (0.663–0.959)		0.799 (0.650–0.981)	
Other	0.658 (0.539–0.802)		0.713 (0.570–0.891)	
Race		0.961		
White	Reference			
Black	0.990 (0.812–1.206)			
Other	0.970 (0.779–1.206)			
Primary site		0.001		0.273
Central	Reference		Reference	
Inner	1.515 (0.994–2.309)		1.176 (0.733–1.886)	
Outer	1.188 (0.797–1.770)		0.997 (0.637–1.561)	
Overlap	1.512 (1.001–2.285)		1.220 (0.769–1.938)	
Unknown	0.970 (0.627–1.500)		0.978 (0.601–1.593)	
AJCC T stage		< 0.001		< 0.001
T1	Reference		Reference	
T2	0.754 (0.617–0.920)		0.821 (0.659–1.022)	
T3	0.423 (0.331–0.541)		0.548 (0.417–0.721)	
T4	0.463 (0.346–0.621)		0.653 (0.469–0.911)	
AJCC N stage		< 0.001		< 0.001
N0	Reference		Reference	
N1	0.532 (0.453–0.626)		0.580 (0.484–0.696)	
N2	0.232 (0.175–0.309)		0.293 (0.214–0.402)	
N3	0.286 (0.209–0.392)		0.351 (0.246–0.501)	
Histology grade		< 0.001		< 0.001
I	Reference		Reference	
II	2.788 (1.776–4.377)		1.827 (1.108–3.011)	
III/IV	5.676 (3.652–8.821)		2.467 (1.499–4.058)	
HR		< 0.001		< 0.001
ER+/PR+	Reference		Reference	
ER+/PR−	2.260 (1.819–2.807)		2.042 (1.610–2.590)	
ER−/PR+	4.050 (2.535–6.471)		3.492 (2.119–5.756)	
ER−/PR−	3.791 (3.194–4.499)		3.059 (2.509–3.729)	
HER‐2		< 0.001		< 0.001
Negative	Reference		Reference	
Positive	2.518 (2.162–2.934)		2.431 (2.054–2.877)	
Histological subtype		< 0.001		0.163
Ductal	Reference		Reference	
Lobular	0.180 (0.112–0.289)		0.605 (0.360–1.017)	
Mix	0.497 (0.333–0.741)		0.934 (0.592–1.473)	
Chemotherapy		< 0.001		0.016
No	Reference		Reference	
Yes	8.312 (4.023–17.171)		2.574 (1.192–5.562)	
Radiotherapy		< 0.001		0.858
No	Reference		Reference	
Yes	0.744 (0.635–0.873)		0.984 (0.820–1.179)	

Abbreviations: CI, confidence interval; HER‐2, human epidermal growth factor receptor 2; HR, hormone receptor; OR, odds ratio.

### 3.3 Nomogram Development and Validation for Predicting Complete Response

**FIGURE 2:**
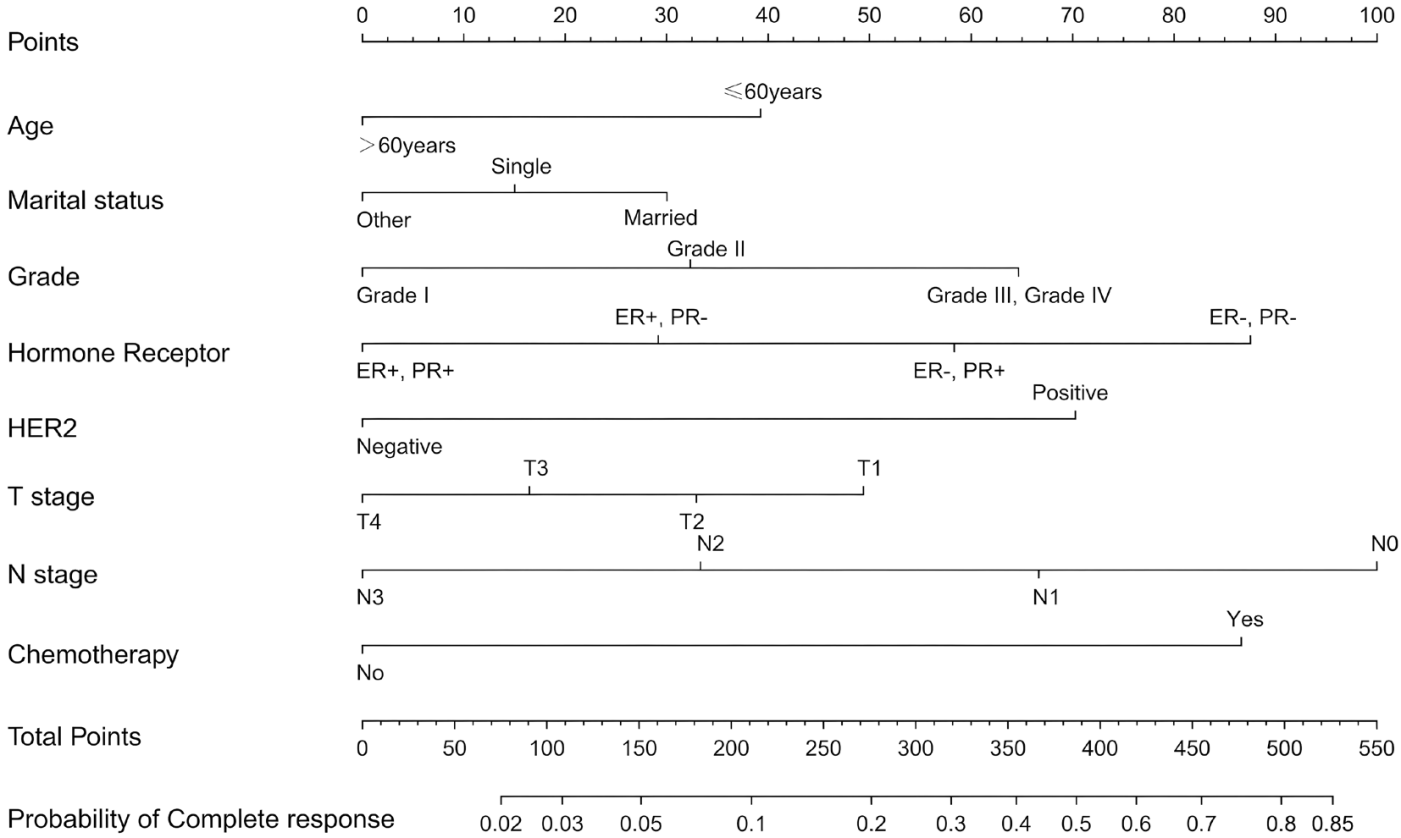
Nomogram for predicting pCR of BC patients in surgery group.

**FIGURE 3:**
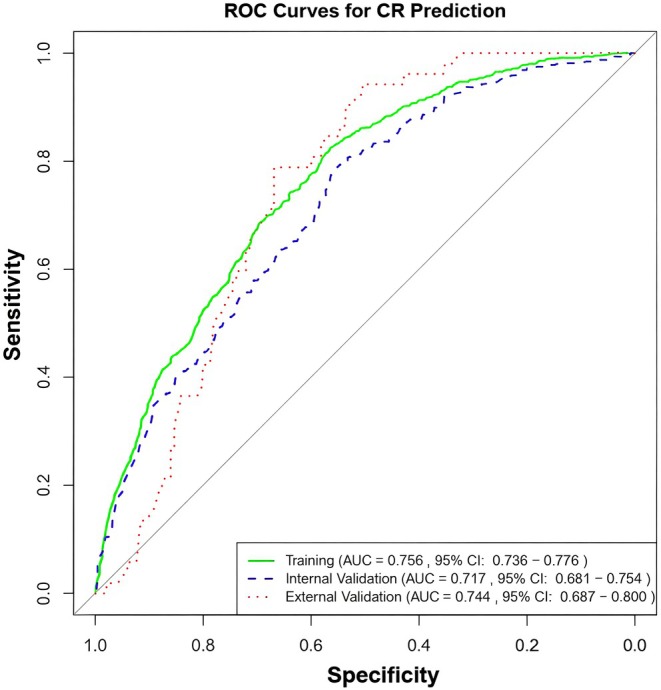
Receiver operating characteristic curves in the training (A), testing (B) and validation (C) groups for pCR.

**FIGURE 4:**
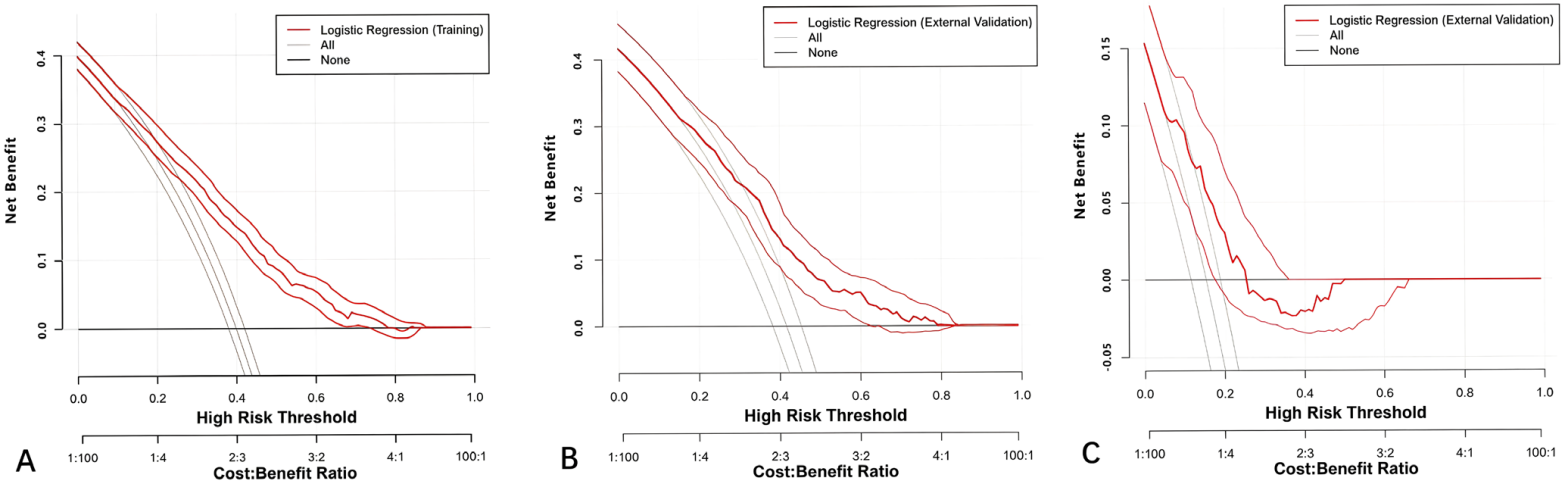
Decision curve analysis curves of the nomogram for pCR of BC patients in the training cohort (A), in the testing cohort (B) and in the validation cohort (C).

A nomogram was developed using eight predictors (Figure 2). The values of the ROC curves were 0.756 (95% CI: 0.736–0.776) in the training cohort (*n* = 2279) and 0.717 (95% CI: 0.681–0.754) in the internal validation cohort (*n* = 758), which was greater than 0.7, reflecting the excellent accuracy and discrimination of the model (Figure 3). The decision curve analysis demonstrated a higher net benefit across a wide range of threshold probabilities compared with the “treat‐all” or “treat‐none” strategies. In the training cohort (Figure 4A), the model provided a clear clinical advantage when the threshold probability ranged from approximately 0.1 to 0.7, indicating that applying the nomogram within this interval would yield a meaningful net benefit for decision‐making. Similar patterns were observed in the internal validation cohort (Figure 4B), with stable performance across thresholds of 0.1–0.6. In the external validation cohort (Figure 4C), the model maintained a modest yet consistent benefit within the 0.1–0.4 threshold range, supporting its potential clinical applicability in independent populations.

### 3.4 Survival Stratification Based on the Nomogram Scores

**FIGURE 5:**
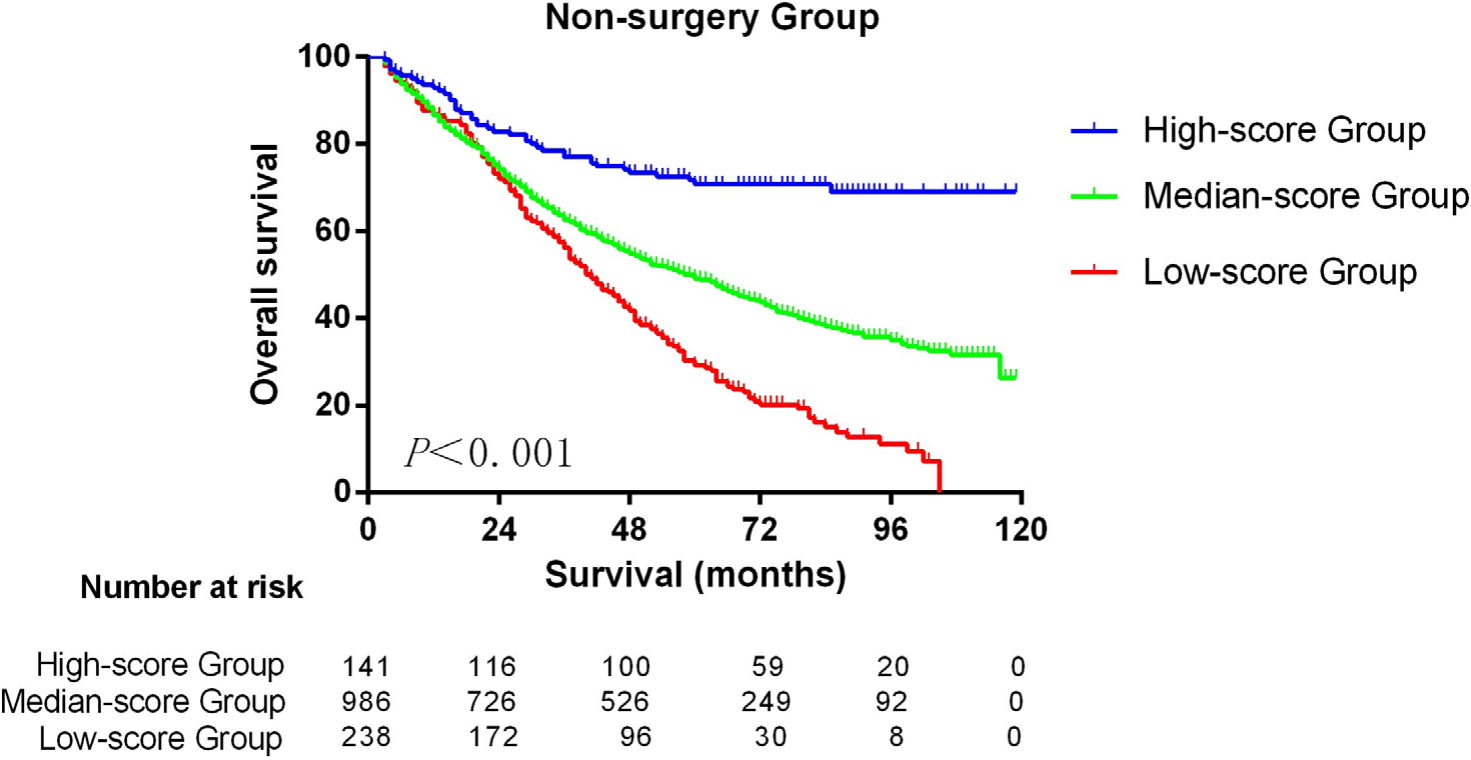
Kaplan–Meier curves of non‐surgery BC patients stratified by nomogram scores.

Non‐surgery BC patients were stratified by nomogram scores: high (> 375, 10.3%), medium (162.5–375, 72.2%), and low (< 162.5, 17.4%). Five‐year OS rates were 70.7% (95% CI: 63.1%–78.3%), 49.2% (95% CI: 46.1%–52.3%), and 29.2% (95% CI: 23.1%–35.3%), respectively (*p* < 0.001, Figure 5). One high‐score patient (score: 482.5) achieved pCR with chemotherapy and radiotherapy, surviving 102 months without surgery.

### 3.5 External Validation

The demographic and clinicopathological characteristics of the 339 BC patients who underwent NAT and surgery from our hospital are shown in Table 3. In the external validation, 80.8% were ≤ 60 years, 99.7% were married. Tumors were predominantly invasive ductal carcinoma, comprising 97.6% of cases, with moderate differentiation in 54.6%, low differentiation in 38.3%, and high differentiation in 7.1%. T stages included T1 (12.1%), T2 (55.2%), T3 (16.8%) and T4 (15.9%), while N stages were N0 (12.7%), N1 (50.1%), N2 (24.8%), and N3 (12.4%). No patients had distant metastasis. Hormone receptor status showed ER+/PR+ in 56.9%, ER+/PR− in 15.3%, ER−/PR+ in 1.8%, and ER−/PR− in 26.0%, with HER2 positivity in 46.3%. Additionally, 15.3% of patients achieved pCR. The nomogram's predictive performance was validated in this cohort, yielding an ROC curve with an AUC of 0.744 (95% CI: 0.687–0.800, Figure 3).

**TABLE 3:** Summary of characteristics for 341 breast cancer patients from our hospital.

Characteristic	Number of patients	Percentage (%)
Age		
≤ 60	274	80.8
> 60	65	19.2
Marital status		
Married	338	99.7
Single	1	0.3
AJCC T stage		
T1	41	12.1
T2	187	55.2
T3	57	16.8
T4	54	15.9
AJCC N stage		
N0	43	12.7
N1	170	50.1
N2	84	24.8
N3	42	12.4
Differentiation grade		
I	24	7.1
II	185	54.6
III/IV	130	38.3
HR		
ER+/PR+	193	56.9
ER+/PR−	52	15.3
ER−/PR+	6	1.8
ER−/PR−	88	26.0
HER‐2		
Negative	182	53.7
Positive	157	46.3
Histological subtype		
Ductal	331	97.6
Lobular	5	1.5
Mix	3	0.9
Complete response		
Yes	52	15.3
No	287	84.7

### 3.6 Comparison of Clinical Outcomes Between Surgery and Non‐Surgery Groups After Propensity Score Matching

**FIGURE 6:**
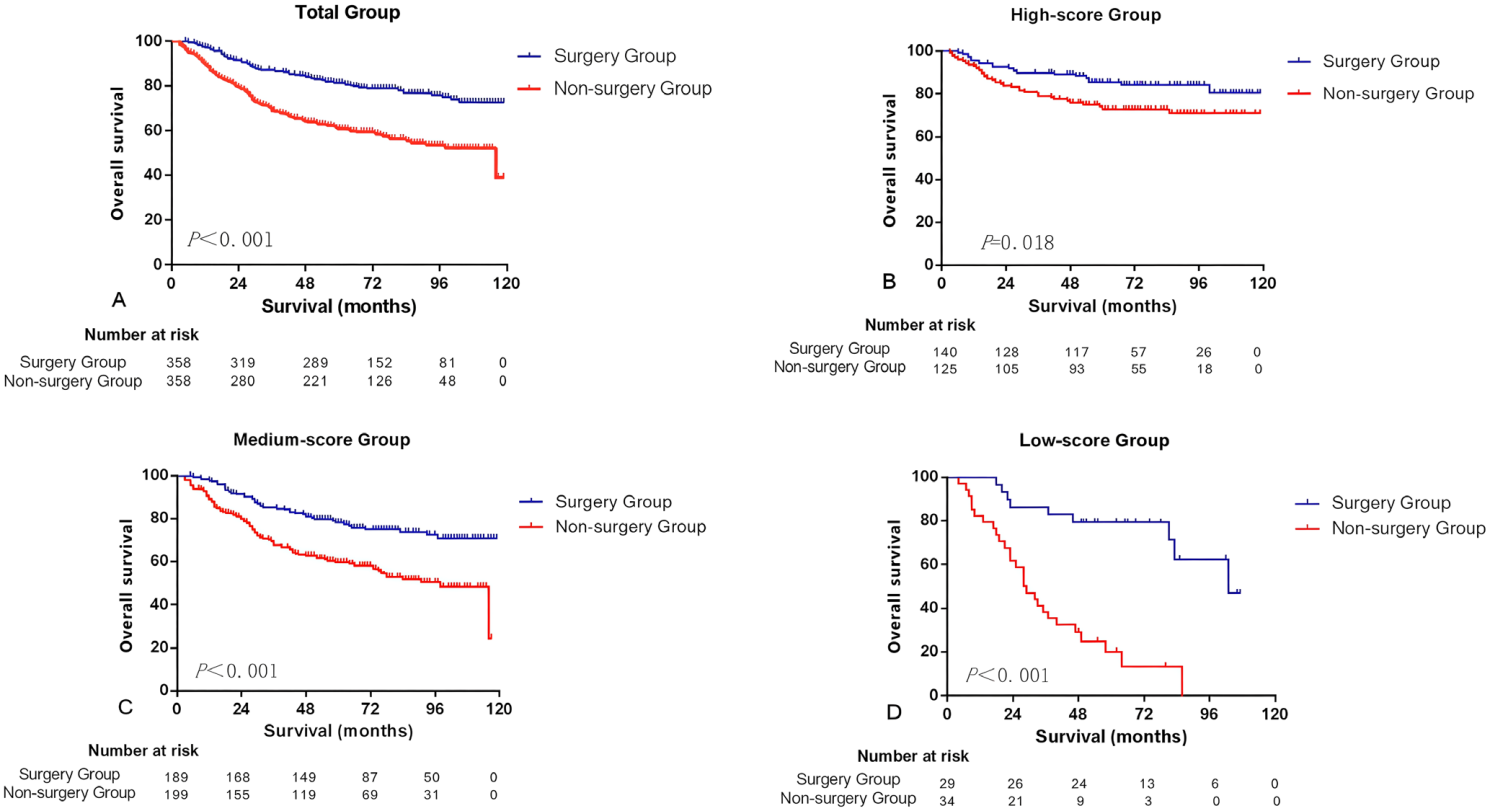
Kaplan–Meier survival analysis for OS according to different nomogram score levels after matching. (A) OS between surgery group and non‐surgery group in total group. (B) OS between surgery group and non‐surgery group in high‐score group. (C) OS between surgery group and non‐surgery group in medium‐score group. (D) OS between surgery group and non‐surgery group in low‐score group.

The matching factors included age, marital status, race, primary site, T stage, N stage, differentiation grade, HR status, HER‐2 status, histological subtype, chemotherapy and radiotherapy. After 1:1 propensity score matching, 358 patients from each group were enrolled in the analysis. No significant differences in baseline characteristics were observed between the two groups after matching (Table 1). Among the matched samples, the 5‐year OS for patients stratified by high‐, medium‐, and low‐score groups was 85.4% (95% CI: 79.3%, 91.5%) vs. 72.8% (95% CI: 64.8%, 80.8%), 78.3% (95% CI: 72.2%, 84.4%) vs. 59.8% (95% CI: 52.7%, 66.9%), and 79.3% (95% CI: 64.6%, 94.0%) vs. 20.0% (95% CI: 5.1%, 34.9%) in the surgery versus non‐surgery groups, respectively (*p* = 0.018, < 0.001, < 0.001) (Figure 6).

## 4 Discussion

This study provided one of the biggest samples for BC patients who received surgery after NAT with accurate drug response and built a reliable nomogram for predicting pCR rate. Our prediction model further indicated that subgroups of BC patients in the non‐surgery group classified into the high‐score group could achieve long‐term survival even without surgery, with a 5‐year OS rate of 72.8%. To my knowledge, this is the first large‐scale study exploring non‐surgical treatments for breast cancer.

This study identified that younger age, married status, earlier T stage, no lymph node metastasis, poor differentiation, ER−/PR−, HER2+, and receiving chemotherapy were more likely to achieve pCR after NAT. Younger patients have better physical ability to tolerate more intensive NAT regimens [13]. Married patients could get more mental and financial support which could help them to get more appropriate treatments [14]. Goorts B et al. reported that lower T stage has a higher pCR rate than higher T stage, which is consistent with our research results [8]. Patients with negative lymph nodes had less tumor burden and were more likely to obtain pCR after NAT. So, negative lymph node status was an independent predictor of pCR in BC patients [15]. Tumors with poor differentiation often proliferate quickly and are more sensitive to drugs. Because cytotoxic drugs could kill cancer cells with high levels of basal proliferation and regeneration [16]. Patients with triple‐negative breast cancer or HR receptor negative/HER‐2 positive had a high probability of pCR, which had been reported in many studies [3, 7, 17, 18]. NAT is more effective for HER‐2 positive breast cancer patients, probably because they are associated with increased tumor infiltrating lymphocytes [19]. Only nearly 10% of ER+ BC patients could get pCR and they were relatively insensitive to chemotherapy compared with other molecular subtypes. Recent studies have also reported that BC patients with ER‐ and PR‐ have a higher probability of pCR [7, 20, 21, 22], which was similar to our results.

Currently, achieving pCR is a critical indicator for potentially meeting the criteria for specific systemic therapies [9]. Ring et al. reported on 136 BC patients who achieved clinical CR, of whom 67 underwent surgery and 69 received radiotherapy without surgery. There was no significant difference in disease‐free survival (DFS) and OS between these two groups (5‐year, 74% vs. 76% and 74% vs. 76%). However, an upward trend in local‐regional recurrence was observed in the radiotherapy group (5‐year, 21% vs. 10%; *p* = 0.09) [23]. Clouth et al. [24] reported that 16 patients assessed as pCR by multiple core needle biopsies received radiotherapy only and others underwent mastectomy or breast‐conserving surgery. There was no difference in DFS or OS, but the overall local recurrence rate was 9.5% for the surgery group and 12.5% for the non‐surgery group. Daveau et al. found that of 165 BC patients who achieved clinical CR after NAT, 65 patients received surgery and radiation therapy, while 100 patients received radiation therapy alone. There were no significant differences in OS and DFS rates between these two groups. Compared with the surgery group, the local control rate of the radiotherapy alone group showed a lower trend (5‐year, 77% vs. 90%, *p* = 0.06) [25]. These studies show that for BC patients who achieved clinical CR, nonsurgical treatments may also be viable options without significantly affecting OS and DFS. It is worth noting that the local control rate of radiotherapy alone may be slightly inferior to that of surgery. However, this may be compensated by increasing the dose of radiotherapy to improve the local control rate. Because the breast is a superficial organ, increasing the dose of radiotherapy is achievable. We hope that there will be prospective clinical trials to explore the appropriate radiotherapy dose in the future.

Most studies on the assessment of NAT response in BC were retrospective, single‐center studies with small samples [26]. Our study with large samples showed that the prognosis of non‐surgery patients in the high‐score group was significantly better than that of the other two groups (5‐year OS: 70.7% vs. 49.2% vs. 29.2%). Compared with the other two groups, patients in the high group had a higher probability of achieving pCR rate from NAT and had better survival. This highlights the practical value of our nomogram in identifying BC patients who can achieve long‐term survival without surgery. Compared with surgery group patients, non‐surgery group patients may lack the necessary examination and treatment. For example, the non‐surgery group had a lower proportion of radiotherapy (26/358, 7.3%). Recent studies have demonstrated significant improvements in the efficacy of NAT and radiotherapy [27, 28]. A better prognosis may be achieved if the proportion of radiotherapy can be increased and effective NAT is adopted for non‐surgery group patients. In our study, one patient achieved pCR with combined radiotherapy and chemotherapy, without surgery. The patient remained alive after 102 months of follow‐up. According to our prediction model, this patient's score is 482.5, which belongs to the high‐score group. This case illustrates the possibility that BC patients achieving pCR combined with radiotherapy and chemotherapy without surgery may be an alternative treatment option. Certainly, this needs to be confirmed by future prospective clinical trials.

Patients in the high‐score group only have a high probability of achieving pCR from systemic treatment, but some may fail to achieve pCR, which is an important factor that reduces OS. Biopsy, ultrasound, MRI, PET‐CT, and other examinations can be used to further improve the accuracy of pCR assessment. In addition, residual ductal carcinoma in situ may also affect prognosis. Most literature defined pCR as invasive disease without residual presence in breast and axillary lymph nodes [7, 8, 22, 29]. The ypT0 ypN0 criterion is more stringent and may be better suited for identifying candidates eligible for surgery omission. Multiple predictive nomograms for pCR in early breast cancer have been reported, yet a systematic review underscored marked heterogeneity across models [30]. Our nomogram provides a useful tool for individualized risk assessment, identifying those who may benefit from treatment intensification or alternative strategies.

This study has several limitations. Firstly, the SEER database lacks important clinical information such as neoadjuvant treatment scheme, genetic testing, Ki‐67, and imaging examination results. Genetic testing should be performed in triple‐negative or HER2‐positive breast cancer, especially for patients who may consider omitting surgery. Secondly, this is a retrospective study, which may introduce bias. Third, the proportion of patients receiving radiotherapy is too low to evaluate its effect. Fourth, the external validation cohort comes from real‐world data and may contain inherent biases, which could limit the generalizability of our model. Therefore, prospective trials are necessary to further verify our conclusions.

## 5 Conclusions

We developed a nomogram to predict pCR in BC patients following NAT. This model identified specific subgroups of BC patients who may achieve long‐term survival without surgery. Further clinical trials are required to determine whether BC patients with pCR receiving radical radiotherapy can safely omit surgery.

## Author Contributions

**Kaining Ye:** writing – original draft. **Xuehong Liao:** methodology. **Weiping Yang:** supervision, resources. **Jianming Weng:** data curation. **Yongliang Dai:** funding acquisition, software. **Xiliang Chen:** validation, investigation, conceptualization. **Yongjian Liu:** validation, conceptualization, investigation. **Kaixin Du:** writing – review and editing, supervision, formal analysis, project administration.

## Ethics Statement

This study was conducted with ethical approval from the Zhangzhou Affiliated Hospital of Fujian Medical University (Ethics Approval Number: 2025‐LWB‐221).

## Consent

The authors have nothing to report.

## Conflicts of Interest

The authors declare no conflicts of interest.

## Data Availability

The SEER dataset is publicly available at https://seer.cancer.gov/. The Zhangzhou cohort data is available from the corresponding author upon reasonable request due to ethical restrictions.
